# Altered Functional Connectivity of Striatum Based on the Integrated Connectivity Model in First-Episode Schizophrenia

**DOI:** 10.3389/fpsyt.2019.00756

**Published:** 2019-10-18

**Authors:** Bei Zhang, Pan Lin, Xiaosheng Wang, Dost Öngür, Xinlei Ji, Weijun Situ, Shuqiao Yao, Xiang Wang

**Affiliations:** ^1^Medical Psychological Center, the Second Xiangya Hospital, Central South University, Changsha, China; ^2^General and Experimental Psychology, Department of Psychology, LMU Munich, Munich, Germany; ^3^Department of Psychology and Cognition and Human Behavior Key Laboratory of Hunan Province, Hunan Normal University, Changsha, China; ^4^Department of Human Anatomy and Neurobiology, Xiangya School of Medicine, Central South University, Changsha, China; ^5^Department of Psychiatry, Harvard Medical School and McLean Hospital, Belmont, MA, United States; ^6^Department of Radiology, The Second Xiangya Hospital, Central South University, Changsha, China

**Keywords:** striatum, meta-analytic connectivity modeling (MACM), resting-state functional connectivity, corticostriatal circuits, schizophrenia

## Abstract

**Background:** The human striatum is a heterogeneous structure involved in diverse functional domains that related to distinct striatum subregions. Striatal dysfunction was thought to be a fundamental element in schizophrenia. However, the connectivity pattern of striatum solely based on functional or structural characteristics leads to inconsistent findings in healthy adult and also schizophrenia. This study aims to develop an integrated striatal model and reveal the altered functional connectivity pattern of the striatum in schizophrenia.

**Methods:** Two data-driven approaches, task-dependent meta-analytic connectivity modeling (MACM) and task-independent resting-state functional connectivity (RSFC), were used for seven anatomical connectivity-based striatum subregions to provide an integrated striatal model. Then, RSFC analyses of seven striatal subregions were applied to 45 first-episode schizophrenia (FES) and 27 healthy controls to examine the difference, based on the integrated model, of functional connectivity pattern of striatal subregions.

**Results:** MACM and RSFC results showed that striatum subregions were associated with discrete cortical regions and involved in distinct cognitive processes. Besides, RSFC results overlapped with MACM findings but showed broader distributions. Importantly, significantly reduced functional connectivity was identified between limbic subregion and thalamus, medial prefrontal cortex, anterior cingulate cortex, and insula and also between executive subregions and thalamus, supplementary motor area, and insula in FES.

**Conclusions:** Combing functional and structural connectivity information, this study provides the integrated model of corticostriatal subcircuits and confirms the abnormal functional connectivity of limbic and executive striatum subregions with different networks and thalamus, supporting the important role of the corticostriatal-thalamic loop in the pathophysiology of schizophrenia.

## Introduction

Recent evidence from nonhuman primates studies (1, 2) and human neuroimaging studies (3) suggests that the striatum, the main recipient of afferents projecting to the basal ganglia, is a complex brain structure with multiple and highly specific functions. It has well-documented roles in motor control and execution (4, 5) and also being involved in a diverse array of other functional domains, such as emotion generation and regulation (6, 7) memory (8, 9), executive functions (10), and reward-related processes (11, 12). These discrete functions appear to be related to distinct corticostriatal circuits, with dissociable striatal subregions receiving different cortical projections (3).

Earlier animal studies have suggested that projections from the cerebral cortex to striatum have a topographic organization, such as rostral areas of cerebral cortex projected to the rostral striatum and caudal areas projected to the caudal striatum (13). However, plenty of subsequent studies indicated that the corticostriatal circuit was more complex than that (14, 15). For example, Ferry et al. (15) in monkeys showed that the ventromedial striatum received input predominantly from the medial prefrontal cortex (mPFC) and some orbital areas, but other areas of orbital cortex projected primarily to the center of the rostral striatum, suggesting that the prefrontostriatal projections reflect the functional organization of the PFC rather than topographic location (15). Autoradiography and molecular positron emission tomography (PET) imaging studies have further demonstrated that dopamine receptors and dopamine release had variable distributions within the traditional topographic anatomical divisions (i.e., caudate, putamen, nucleus accumbens) (16–18). All the above evidence indicated the functional heterogeneity within traditional topographic and anatomical-based striatal subregions.

Gradually, functional subregions had been introduced according to the distribution of corticostriatal inputs. For example, Alexander and colleagues proposed a striatum model composed of five segregated and parallel functional loops. In their model, each definable striatal area receives input from a particular cortical area and sends efferent to specific basal ganglia nuclei that, ultimately, project back to the same part of the cortex by way of the thalamus (19). Besides, Haber (20) was able to divide the striatum into three subregions—limbic striatum, associative striatum, and sensorimotor striatum—according to afferent inputs from the frontal cortex, which themselves appear to be organized in a hierarchical manner and divided into limbic, associative, and motor functional subregions. However, it has been a challenge to delineate the topographical organization of these functional subregions, especially concerning the traditional anatomical subregions of the striatum (21). Hence, integrating the functional and structural characteristics of the striatum remains an unresolved problem.

In the last two decades, investigators began to examine functional and structure striatal subregions and corticostriatal circuitry of the human brain *in vivo* with noninvasive neuroimaging methods, such as task-based functional protocols and intrinsic functional connectivity analyses (22–26), diffusion tensor imaging (DTI) (27–29), and T1-weighted voxel-based morphometry (30). However, most of previous researches exploring the connectivity of the human striatum used either traditional topographic anatomical divisions (i.e., caudate, putamen, nucleus accumbens) (23, 24) or atlas from animal studies (28) as regions of interests (ROIs) with a single imaging technology. Importantly, recently Tziortzi and colleagues parcellated the striatum into seven subregions using both the structural connectivity-based method and the functional molecular imaging method (31). Specifically, in this pioneering work, they first used the probabilistic tractography method in diffusion magnetic resonance imaging (MRI) data to segment striatum based on the calculation of voxel-wise probability in the whole striatum to predefined target regions of the cerebral cortex. From that, the striatum was subdivided into seven structural connectivity-based subregions. Further, they quantitatively measured local neurotransmitter releasing with PET data and demonstrated significantly higher homogeneity of dopamine release within their structural connectivity-based subregions than within traditional structural subregions, thereby providing a basis from which to explore the functional specialization of the striatum.

Striatal dysfunction has long been thought to be a fundamental element in schizophrenia in the different hypothesis of the etiology of schizophrenia, no matter its neurochemical dopamine hypothesis (32), or the neurodevelopmental hypothesis (33), or the disconnection hypothesis (34). For example, schizophrenia is considered as dopamine dysregulation in striatum and disruptions in corticostriatal circuitry in the dopamine hypothesis related to typical antipsychotics. Previous researchers revealed stable abnormal functional connectivity of striatum, especially in corticostriatal circuitry. Liang et al. (35) found a comprehensive decreased functional connectivity in schizophrenia throughout the entire brain during rest, specifically in the insula, the temporal lobe and the prefrontal lobe, and the striatum. Koch et al. (36) further indicated reduced functional connectivity between left striatum and temporo-occipital areas, precuneus, and insula in the schizophrenia. A follow-up and treatment study showed corticostriatal dysfunctional connectivity and suggested the increased functional connectivity of the striatum with prefrontal and limbic regions could be a biomarker for improvement in symptoms (37). However, the examination of the functional connectivity of the striatum in schizophrenia has either taken the striatum as a whole part, or based on ROI of striatum from animal studies, or based on traditional structure subregions; combing the confounding effect from the method of analysis, medications, and heterogeneous sample, the functional connectivity impairments of striatum in schizophrenia are still unclear.

Meta-analytic connectivity modeling (MACM) is a large-scale, unbiased, task-dependent, and data-driven approach to generate a precise, comprehensive functional connectivity map (38). Rather than relying on prior assumptions, MACM enables task-based functional connectivity meta-analysis across a large number of functional neuroimaging experiments unrestricted by the sample size, sample characteristics, experimental paradigm, and analysis methods of included studies. The basic logic of MACM is that functional connectivity results in coactivation of the brain areas during the performance of relevant tasks. Activation peaks from task-based neuroimaging studies are used to identify consistent (above chance) coactivation across comprehensive coordinates to indicate functional connectivity between brain areas. To date, MACM has been used to explore the functional connectivity of several key brain areas, such as the amygdala (38), insula (39), and cingulate cortex (40), as well as to explore the subregions within a brain region, such as the ventral and dorsal subregions of mPFC (41). Besides, resting-state functional connectivity (RSFC) analysis is also a data-driven but task-independent methodology that has undergone rapid advancement over the last two decades. The RSFC provides an opportunity to monitor fluctuations in the whole brain simultaneously for spontaneous brain activities and quantify the temporal correlations between two brain regions spatially separated. Recently, neuroimaging studies also identified various principal intrinsic brain networks that are activated differently and that present dynamic alterations and switches between a resting state and task stages (42).

Therefore, this study aims to combine the task-dependent MACM and task-independent RSFC approaches to seven anatomical connectivity-based striatum subregions described by Tziortzi et al. (31) to enable an integrated template of the striatum. Furthermore, based on the integrated striatal model, we examine the dysfunctional connectivity of striatum in first-episode schizophrenia (FES) to explore the specific alternations of the striatum subregions and its related cortical pathways to provide new evidence for the pathophysiology and etiology of schizophrenia.

## Methods and Materials

### Seed Regions

**Figure 1:**
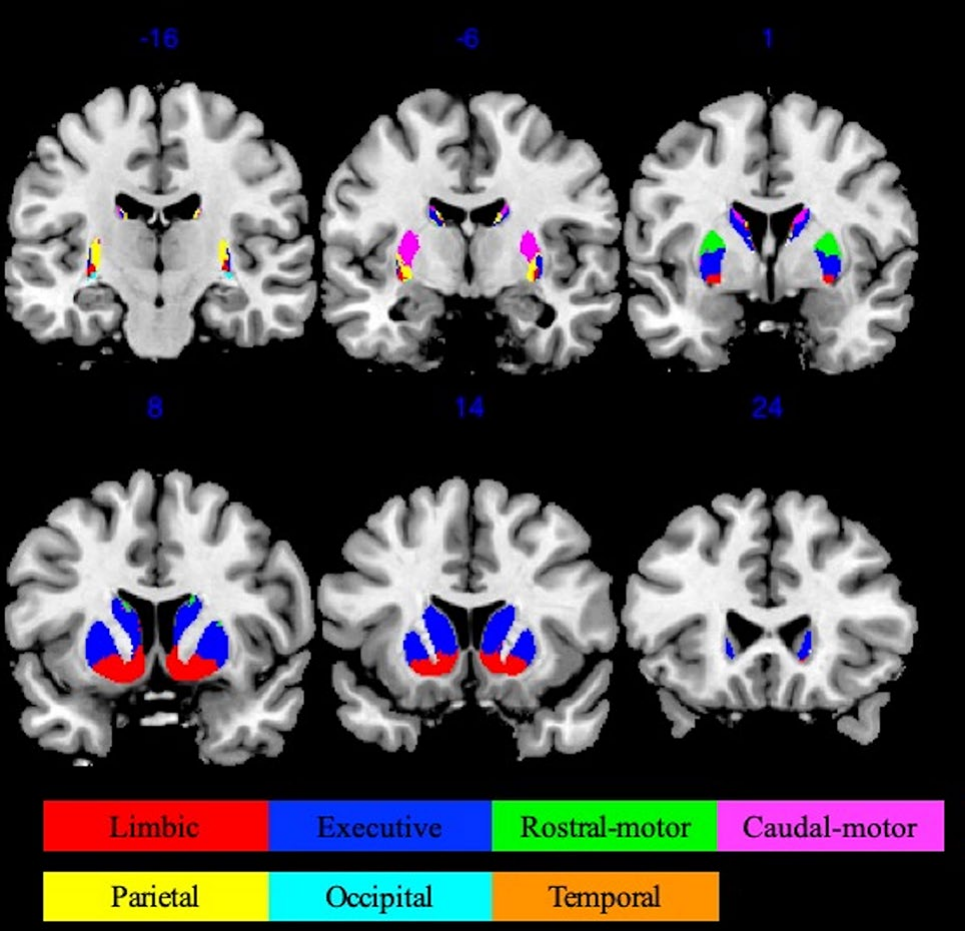
Oxford-GSK-Imanova striatal connectivity atlas (adapted from Tziortzi et al. (31) , https://fsl.fmrib.ox.ac.uk/fsl/fslwiki/Atlases/striatumconn). This probabilistic connectivity striatal atlas was segmented into seven subregions (seven striatum subregions: limbic, executive, rostral-motor, caudal-motor, parietal, occipital, and temporal) according to cortical-striatal anatomical connections. The atlas we used here was based on a threshold of 50% containing 90 voxels at 1 × 1 × 1 mm^3^ resolution.

Striatal ROIs were defined based on the Oxford-GSK-Imanova connectivity striatal atlases (http://fsl.fmrib.ox.ac.uk/fsl/fslwiki/Atlases/striatumconn). We adopted Tziortzi and colleagues’ (31) connectivity atlas based on their study of corticostriatal structural connections by way of multimodal imaging, which was composed of the following resultant seven striatum subregions: limbic, executive, rostral-motor, caudal-motor, parietal, occipital, and temporal. We used the following respective abbreviations to avoid confusion: Str_limbic, Str_executive, Str_rostral-motor, Str_caudal-motor, Str_parietal, Str_occipital, and Str_temporal. The 50% threshold atlases (90 voxels at 1 × 1 × 1-mm^3^ resolution) were used to provide maximal accuracy in our connectivity descriptions (**Figure 1**).

### First MACM Analysis

MACM identifies brain regions that are coactivated above chance with a particular ROI across a large number of functional neuroimaging experiments (38). It is based on the fact that functional imaging studies are normally presented in a highly standardized format (i.e., standard coordinate systems) in large-scale databases, such as BrainMap (43). For our MACM, as is typical, we first identified reports in the BrainMap database showing neural activation within each striatal ROI. Only studies reporting task-based activations in healthy subjects were considered eligible for inclusion. Between-group contrasts, studies involving patient populations, and intervention studies were excluded. Second, we collated the reported foci, sample sizes, Brainmap-delineated behavior domains (BDs) (i.e., action, cognitive, emotion, interception, and perception; https://brainmap.org/taxonomy/behaviors.html), and Brainmap-delineated paradigm classes (PCs) (https://brainmap.org/taxonomy/paradigms.html) for each striatal ROI. Third, we employed the revised activation likelihood estimation (ALE) algorithm, which uses a random-effects model and is weighted for the sample size of the original experiments (44), to test for interstudy convergence of foci within each striatal ROI (45). The activation foci reported in the original studies were treated as three-dimensional Gaussian distributions centered on the reported ALE coordinates. Finally, we calculated the activation probabilities for each standard-space voxel to construct ALE maps for contrasts of interest (44, 46, 47). The ALE maps reported in this study reflect a convergence of coactivations subjected to false discovery rate (FDR) correction with a statistical signiﬁcance threshold of *p* < 0.01. Cortical brain regions found to have coactivation with each striatal ROI are reported with Brodmann area (BA) specifications.

### MRI Data Acquisition

#### Subjects

Forty-nine participants with the FES were recruited from the Department of Psychiatry of the Second Xiangya Hospital of Central South University, Changsha, China. All participants were diagnosed with schizophrenia using the Structural Clinical Interview for *Diagnostic and Statistical Manual of Mental Disorders, Fourth Edition* (*DSM-IV*), Patient Version, and met the following inclusion criteria: (1) met the *DSM-IV* criteria for schizophrenia; (2) first episode, without receiving antipsychotics drugs or any schizophrenia treatment (including benzodiazepines drugs and other sedative-hypnotics); (3) 16 to 40 years of age and had a duration of illness ≤2 years; (4) education duration no less than 9 years; (5) the Han Chinese, right hand; and (6) understand the research and be able to complete the study. Participants were excluded if they had (1) a history of neurological illness or other serious physical illness; (2) a history of substance-related disorders; (3) a history of electroconvulsive therapy; (4) a contraindication to MRI; (5) IQ <70. In the end, 45 FESs were adopted in analysis, and four patients were eliminated for data quality or head movements.

Thirty-one healthy adult volunteers were recruited aged between 16 and 40 years, right-handed, free from clinically significant illness, current or previous history of neurological or psychiatric diagnosis, and alcohol or drug addiction. Individuals with a family history of psychiatric illness among their first-degree relatives were also excluded from the healthy group. In the end, 27 healthy controls were adopted in analysis, and four were eliminated for data quality or head movements.

Demographic and clinical characteristics of participants in the two groups were compared using χ^2^ tests for categorical variables and independent-samples *t* tests for continuous variables. Statistical analyses were performed using the Statistical Package for Social Sciences, version 17.0 (SPSS Inc., Chicago, IL, USA).

Informed consent was obtained from all subjects, and the study obtained the approval of the Institutional Ethical Board of the Second Hospital of Xiangya, Central South University.

#### Data Acquisition

Data were acquired on a 3.0-T Intera Achieva X (Phillips, Holland) whole-body MRI system equipped with a 20-channel Head Matrix Coil. To help stabilize head position, each subject was fitted with a thermoplastic mask fastened to holders on the head coil. During functional scans, subjects viewed a black background and were instructed to relax, stay still, stay awake, and keep their eyes open.

Three-dimensional structural MRI images (T1-weighted) were acquired from the sagittal plane using spoiled gradient echo pulse sequence, with scanning parameters: repetition time (TR) = 8.5 ms, time to echo (TE) = 3.743 ms, flip angle = 8°, field of view (FOV) = 256 × 256 mm, matrix = 256 × 256, voxels = 1 × 1 × 1 mm, slices number = 180, slice thickness =1 mm, gaps = 0 mm.

Resting-state functional images were obtained using a blood oxygenation level–dependent (BOLD) contrast-sensitive gradient echo echo-planar sequence, and we acquired 206 images in total, with scanning parameters: TR = 2,000 ms, TE = 30 ms, flip angle = 90°, FOV = 240 × 240 mm, matrix = 64 × 64, voxels = 3.75 × 3.75 × 4 mm, slices number = 36, slice thickness = 4 mm, gap = 0 mm.

### MRI Data Processing and Analysis

#### Preprocessing

Data were processed in SPM8 (University College; London, UK; http://www.fil.ion.ucl.ac.uk/spm/software/spm8/) and DPABI (48, http://rfmri.org/dpabi) based on MATLAB R2016. Prior to further processing, the first six echo-planar imaging (EPI) images were discarded from the total of 206 images to remove the influence of the shimming coil and adaptation of subjects. The remaining 200 EPI images were corrected for interslice time differences for every subject first. Then, the EPI images were corrected for head movement by affine registration in a two-pass procedure realigning EPI volumes to its mean image. Subjects who had translations head motion of more than 2 mm or rotations of more than 2 degrees were excluded in further analysis. Subsequently, the mean EPI image for each subject was spatially normalized to a standard Montreal Neurological Institute template using the “unified segmentation” approach, and the ensuring deformation field was applied to the individual EPI volumes. The resultant images were smoothed with a 6-mm full-width-at-half-maximum Gaussian kernel to improve the signal-to-noise ratio and to compensate for residual anatomical variations. After normalization, the data were detrended to remove the linear trend and nuisance covariates, including 24 head motion parameters from derived image realignment, global mean signals, white matter signals, and cerebrospinal fluid signals, and were regressed out of the signals to reduce the risk of spurious correlations. Finally, band-pass–filtered preserving BOLD frequencies were between 0.01 and 0.08 Hz.

#### Functional Connectivity Analysis

For each subject, functional analysis was performed between each striatal ROI and the rest of the brain in a voxel-wise manner. To improve normality, the correlation coefficients in each voxel were transformed to *z* values by way of the Fisher *r*-to-*z* transformation.

1. RSFC analysis of HC. One-sample *t* tests and general linear modeling in SPM8 were applied to *z* value maps for healthy control group to obtain functional connectivity maps of the seven striatal ROIs separately. Results were reported at a height threshold of *p* < 0.001(uncorrected) and an extent threshold of *p* < 0.05 (family-wise error rate corrected) with a minimum cluster size of 10 contiguous voxels.
2. Conjunction analysis of MACM and RSFC. To delineate areas showing task-dependent and task-independent connectivity with striatum subregions, we performed MACM-RSFC conjunction analysis with strict minimum statistics (49). For each seed region, we identified those voxels that showed significant connectivity with that seed in an interaction analysis in the task-dependent and task-independent state.
3. RSFC analysis between FES and HC. For each striatal subregion, two-sample *t* tests were used to *z* value maps between the schizophrenia group and the healthy control group to contrast significant difference of functional connectivity patterns. Results of the above conjunction analysis of MACM and RSFC for each seed region were set as a mask while conducting the *t* tests, at a height threshold of *p* < 0.001(uncorrected) and an extent threshold of *p* < 0.05 (FDR corrected).

## Results

### MACM Analysis

#### Article Inclusion

A total of 1,023 studies published no later than May 31, 2018, were included in our meta-analysis. These studies corresponded to 1,366 experiments and 2,976 experimental conditions, with a total of 16,369 subjects and 22,026 activation locations. Detailed descriptions of the striatal ROIs can be found in **Table 1**. For example, the Str_limbic subregion was identified in 250 papers, 358 experiments, and 728 conditions in the Brainmap database, with data for 4,277 subjects and 4,915 locations being subjected to further ALE analysis.

**Table 1:** The number of articles, experiments, conditions, subjects, and locations identified by each striatal subregion in the Brainmap database.

Striatal subregions	Papers	Experiments	Conditions	Subjects	Locations
Str_limbic	250	358	728	4,277	4,915
Str_executive	484	676	1,450	7,888	11,107
Str_rostral-motor	80	93	223	1,242	1,850
Str_caudal-motor	115	135	311	1,543	2,335
Str_parietal	74	84	202	1,113	1,447
Str_occipital	16	16	54	228	236
Str_temporal	4	4	8	78	136

For simplicity, the abbreviations Str_limbic, Str_executive, Str_rostral-motor, Str_caudal-motor, Str_parietal, Str_occipital, and Str_temporal were adopted wherein Str means striatal.

#### Functional Connectivity Model

**Figure 2:**
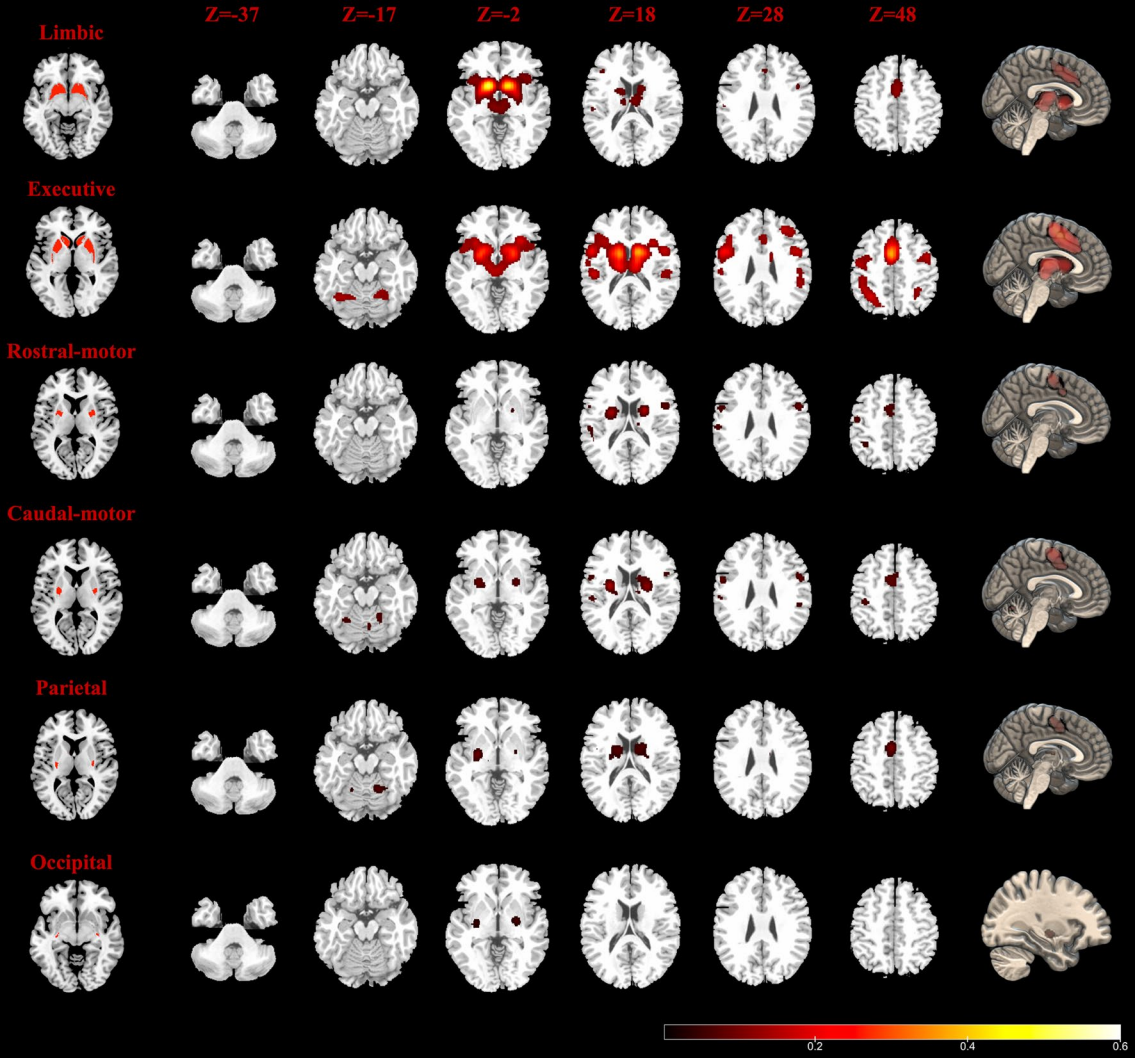
Task-dependent meta-analytic connectivity modeling (MACM) results for the seven striatum subregions. The result of temporal subregion was not demonstrated because of no significant connectivity at the current threshold. The first column (left): six striatum subregions (ROIs); the last column (right): connectivity modeling results of six striatum subregions (FDR correction with a statistical threshold of *p* < 0.01); middle columns: axial plane views (at *z* = −37, −17, −2, 18, 28, 48) of connectivity results at the right side.

The MACM results are shown in **Figure 2**. The Str_limbic subregion was found to be functionally connected with several subcortical and frontal cortex regions (details in **Table S1**), including the bilateral thalamus (medial dorsal nucleus), left putamen, right caudate, bilateral insula (BA13), bilateral cingulate gyrus (BA24/32), left mPFC (BA6), and left precentral/postcentral gyrus (BA44/40). In addition to having connectivity with the bilateral thalamus (medial dorsal nucleus), insula (BA13), and putamen, the Str_executive subregion (**Figure 2** and **Table S2**) also showed strong functional connectivity with areas in the frontoparietal cortex, including the bilateral middle frontal gyrus (BA6/9/46), inferior frontal gyrus (IFG) (BA9), left mPFC (BA6), bilateral precentral gyrus (BA4/6/44), postcentral gyrus (BA40), inferior parietal lobule (BA7/40), and left superior parietal lobule (BA7). Additionally, the bilateral superior temporal gyrus (STG) (BA22/41/42) and cerebellum were active together with the seed region.

The Str_rostral-motor subregion (**Figure 2** and **Table S3**) showed strong functional connectivity with subcortical areas including the putamen and the ventral posterior lateral nucleus in the left thalamus, as well as functional connectivity with a number of areas in the cerebral cortex, including the bilateral precentral gyrus (BA4/6), right mPFC (BA6), bilateral IFG (BA9/44), left cingulate gyrus (BA24), right STG (BA22), left inferior parietal lobule (BA7/40), left postcentral gyrus (BA2/40), and insula (BA13). Our findings for the Str_caudal-motor subregion (**Figure 2** and **Table S4**) were in part similar to those of the Str_rostral-motor, including strong connectivity with the bilateral thalamus (ventral posterior lateral nucleus), bilateral putamen, left insula (BA13), bilateral IFG (BA9), and left mPFC (BA6), as well as with the bilateral precentral gyrus (BA4/6/44), left postcentral gyrus (BA3/40), right cingulate gyrus (BA24), and left inferior parietal lobule (BA40).

Activity of the Str_parietal subregion (**Figure 2** and **Table S5**) was coincident with activity in the bilateral ventral posterior lateral and medial dorsal nuclei of the thalamus as well as activity in the bilateral putamen, right caudate body, left mPFC (BA6), right cingulate gyrus (BA24), right insula (BA13), and bilateral cerebellum areas. Meanwhile, only the left putamen and right lateral globus pallidus connectivity with the Str_occipital were found to have activity indicative of functional subregion (**Figure 2** and **Table S5**); no regions showed significant functional connectivity with the Str_temporal subregion (**Table S5**).

#### BDs and PCs

**Figure 3:**
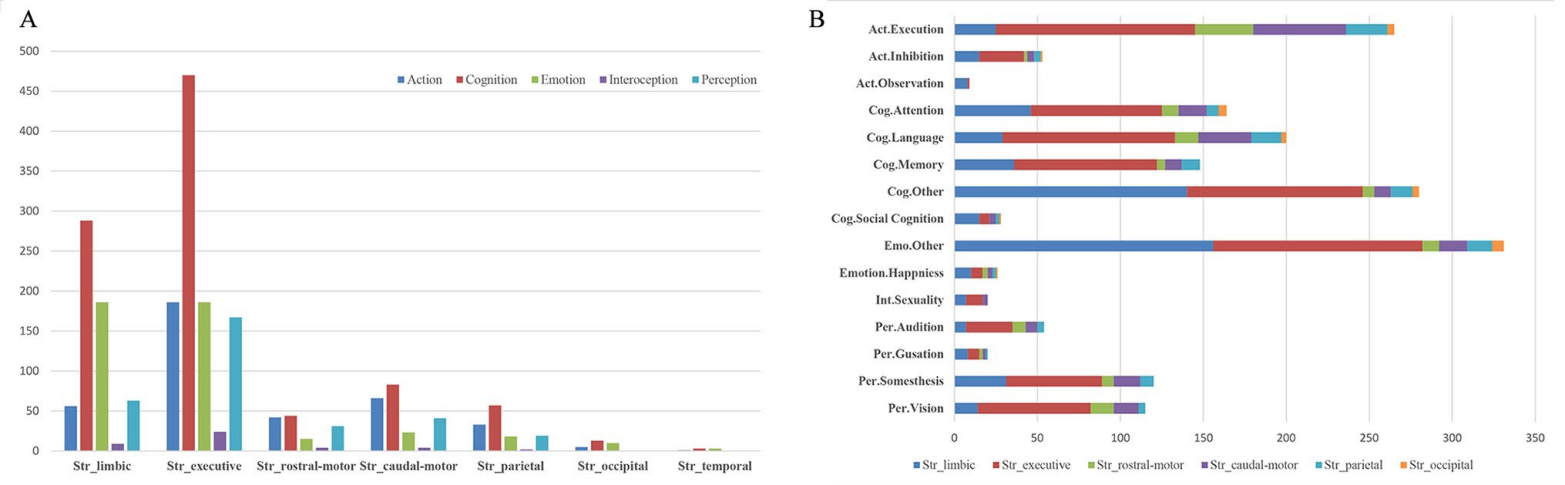
Behavior domains (BDs) and their subcategories associated with each striatal subregion in MACM analysis. **(A)** (left): histograms of the Brainmap-delineated BDs for each striatal subregion; **(B)** (right): the top 15 subcategories of BDs for all seven striatal subregions.

**Figure 4:**
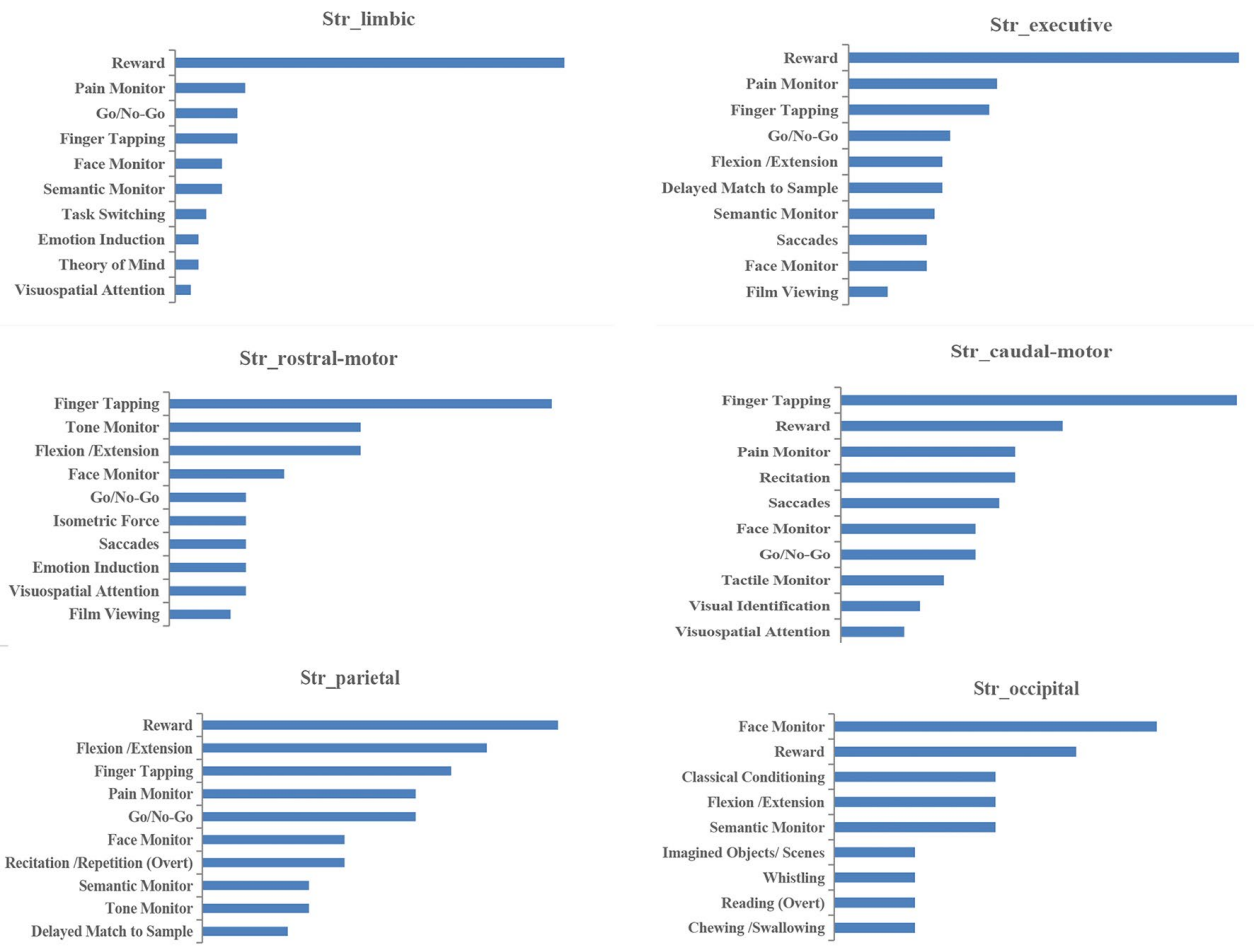
Paradigm classes (PCs) associated with each striatal subregion in MACM analysis.

Histograms of the BDs and its top 15 subcategories for each striatal ROI in our MACM are presented in **Figure 3**. Also, the top 10 PCs for each striatal ROI are presented in **Figure 4**. Analysis of BDs overrepresented among experiments showing regional coactivation with the Str_limbic subregion revealed significant meta-data labels related to cognition and emotion, as well as a significant association with reward-related PCs. BDs that were overrepresented among experiments showing regional coactivation with Str_Executive were also related to cognition, especially for the subcategories of explicit memory and language. PCs significantly associated with Str_executive coactivation included reward and pain monitoring.

BDs that were overrepresented among experiments showing regional coactivation with both the Str_rostral-motor and Str_caudal-motor subregions were action (especially for the execution subcategory) and cognition (especially for the language and attention subcategories). PCs related to Str_rostral-motor coactivation were primarily finger tapping, tone monitoring, and flexion/extension; PCs related to Str_caudal-motor coactivation were finger tapping, reward, and pain monitoring.

BDs that were overrepresented among experiments showing regional coactivation with the Str_parietal subregion were cognition and action. PCs that had significant associations with Str_parietal were reward, flexion/extension, finger tapping, pain monitoring, and go/no-go. BDs that were overrepresented among experiments showing regional coactivation with Str_occipital were cognition, emotion, and action. PCs related to Str_occipital were primarily face monitoring and reward.

### Conjunction Analysis of MACM and RSFC

**Figure 5:**
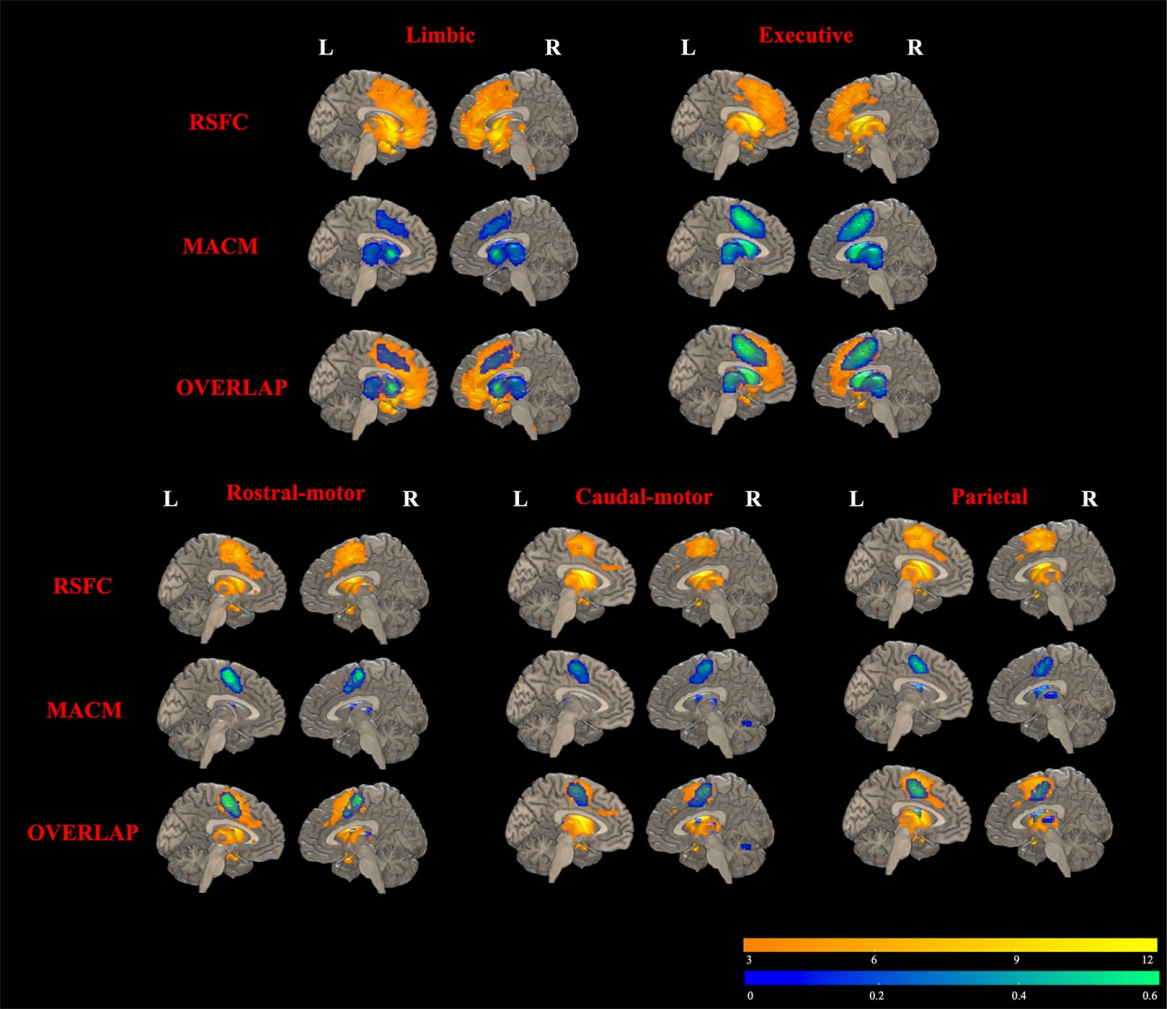
Overlapping results of task-dependent MACM and task-independent RSFC for the seven striatal subregions. Due to few/no significant connectivity results of occipital/temporal striatum subregion in MACM analysis at the current threshold, only functional connectivity modeling of five striatal subregions is presented. RSFC results are depicted in yellow in the top rows (FDR correction with a statistical threshold of *p* < 0.01). MACM results are displayed in blue in the middle rows (FDR correction with a statistical threshold of *p* < 0.01). Overlapped areas of yellow and blue in the bottom rows represent the consistent results of RSFC and MACM.

Results of RSFC of healthy controls were detailed as described in Supplementary Material (Results: 2. Task-independent RSFC analysis). As shown in **Figure 5**, MACM-RSFC conjunction analysis revealed substantial overlaps between our MACM results and the Str_limbic, Str_executive, Str_rostral-motor, Str_caudal-motor, and Str_parietal networks revealed by our RSFC analysis.

### Between-Group Differences of HC and FES

The demographic and clinical data from the 45 patients and 27 healthy controls are shown in **Table 2**. There were no significant differences between the FES group and the HC group in age (*t* = 1.207, *p* = 0.231) or gender (χ^2^ = 1.000, *p* = 0.596).

**Figure 6:**
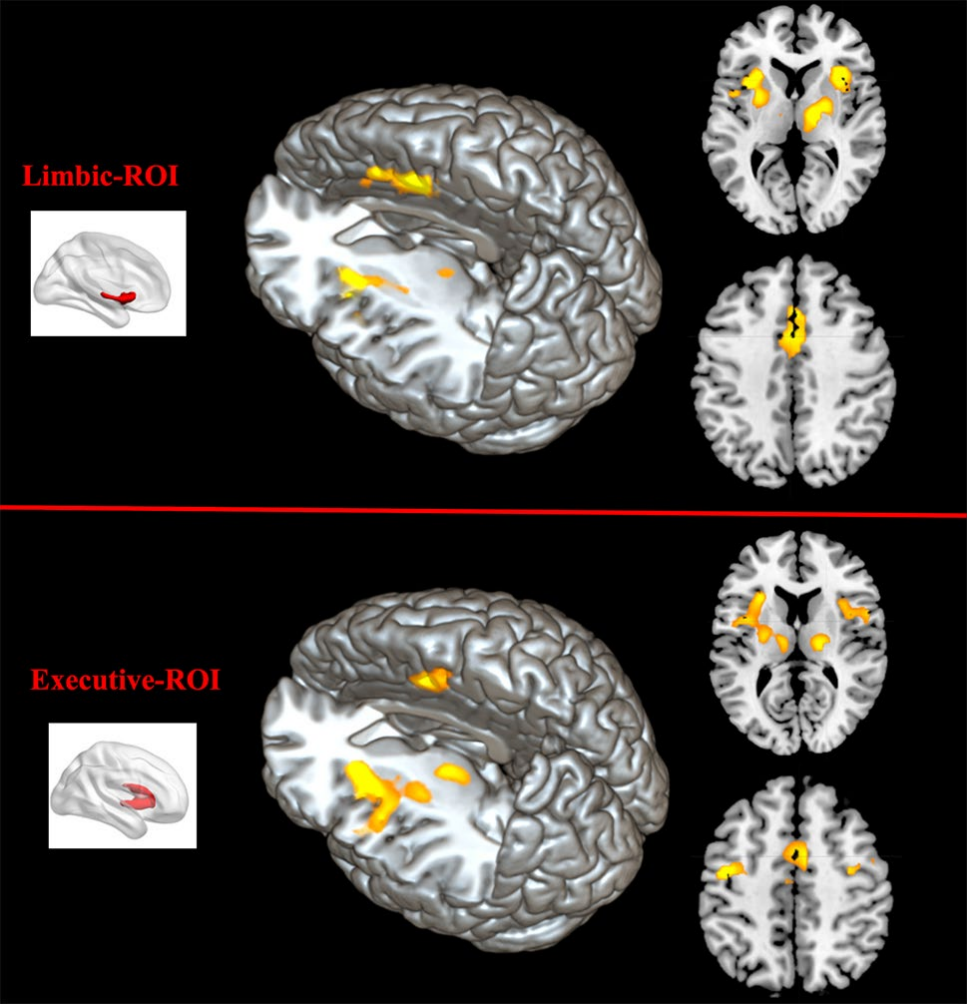
Between-group functional connectivity difference of limbic and executive striatal subregions in HC and FES. Comparing with HC, patients with FES showed reduced functional connectivity between limbic striatum subregion (top region) and thalamus, mPFC, ACC, IFG, and insula (yellow areas with FDR corrected and an extent threshold of *p* < 0.05), as well as between executive subregion (bottom region) and thalamus, SMA, and insula (yellow areas with uncorrected and a height threshold of *p* < 0.001).

Comparing to HC, FES group showed weaker RSFC between Str_limbic and bilateral anterior cingulate gyrus (ACC, BA24/32), mPFC (BA32), left insula, and also right thalamus and left putamen (FDR corrected at the whole brain voxel level, with a significance threshold set at *p* < 0.05; **Table 3** and **Figure 6**). Besides, FES also showed reduced functional connectivity between Str_executive and right thalamus, bilateral insula, right supplementary motor area (SMA), left cingulate gyrus, and precentral areas (uncorrected, with a significance threshold set at *p* < 0.001; **Table 3** and **Figure 6**). There was no significant functional connectivity difference between HC and FES in rostral-motor, caudal-motor, parietal, occipital, and temporal of striatal subregions.

**Table 2:** Demographic and clinical variables.

	Schizophrenia (n = 45)	Healthy controls (n = 27)	Statistical test	
			*t*/χ^2^	*p*
Age (years)	21.31 ± 5.50	22.56 ± 3.25	1.207	0.231
Sex (male/female)	25/20	15/12	1.000	0.596
Duration of illness (month)	10.98 ± 8.09			
PANSS sum-score	81.70 ± 11.87			
Positive sum-score	20.95 ± 6.39			
Negative sum-score	19.89 ± 9.05			
General psychiatric	40.86 ± 7.91			

**Table 3:** Between-group functional connectivity analysis in HC and FES.

Cluster	Anatomical Region	BA	X	Y	Z	Volume (mm^3^)	*t*
Str_limbic							
Control > schizophrenia^1^							
1	L medial frontal gyrus		−6	−3	51	69	3.89
	R medial frontal gyrus	32	6	3	48		3.67
	L cingulate gyrus	24	−6	6	39		2.78
2	R insula		33	18	6	40	3.67
3	R thalamus		15	−12	6	63	3.65
4	L putamen		−30	3	0	77	3.42
	L insula		−36	12	3		3.21
5	R cingulate gyrus	32	3	21	42	1	2.93
6	R anterior cingulate		3	6	−6	1	2.82
Str_executive							
Control > schizophrenia^2^							
1	L precentral		−45	−6	48	16	4.08
2	L insula		−30	18	9	20	3.97
3	L cingulum_mid		−9	6	33	5	3.95
4	R supp_motor_area		6	3	46	4	3.52
5	R thalamus (ventral lateral nucleus)		15	−18	12	8	3.49
6	R insula		36	15	6	3	3.45

^1^Results were reported at a height threshold of p < 0.001(uncorrected) and an extent threshold of p < 0.05 (FDR corrected).

^2^Results were reported at a height threshold of p < 0.001(uncorrected).

## Discussion

The present study examines the dysfunction connectivity of striatum in FES based on the integrated functional model of the striatum that arises from the functional connections of the seven structural connectivity-based striatum subregions. Our functional connectivity model results for the corticostriatal subcircuits of these seven subregions were highly consistent with the structural connectivity evidence that was used to subdivide the striatum originally, especially for subregions connected with the PFC, such as Str_limbic, Str_executive, Str_rostal-motor, and Str_caudal-motor. We further observed considerable overlap between task-dependent MACM results and task-independent RSFC analysis results. More importantly, abnormal functional connectivity of limbic and executive of striatum subregions was identified in schizophrenia.

### Consistent Striatocortical Functional and Structure Connectivity

The striatal subregions adopted in this study were derived from Tziortzi and colleagues’ (31) DTI and probabilistic tractography study, which demonstrated structural connectivity between the particular cerebral cortex regions and striatal subregions. Specifically, Str_limbic subdivision is mainly connected with orbital gyrus and mPFC/ventral anterior cingulate (BA24); Str_executive subdivision is connected with dorsolateral PFC (BA9, BA9/46, and BA10); Str_rostral-motor subdivision is connected with pre–supplementary motor area (BA6); and Str_caudal-motor subdivision is connected with primary motor cortex (BA4) and caudal premotor area (BA6) in the precentral gyrus. The MACM results in the current study were generally consistent with the aforementioned structural connectivity, as well as showed corresponding significant task-related functional connectivity.

In our MACM analysis results, limbic subregion showed significant functional connectivity with mPFC/anterior cingulate (BA24/32) and ventrolateral PFC/IFG (BA47). The corresponding BD and PC analysis showed those coactivations were related mainly to the emotion and cognition domain, and the performance of reward task paradigms. And the Str_executive subregion was found to have widespread functional connectivity with the cortex, especially with the frontoparietal cortex. Again, the BD and PC analysis showed paradigms that are dependent upon explicit memory and language. Both motor subregions, Str_rostral-motor and Str_caudal-motor, shared similar functional connectivity. However, Str_rostral-motor coactivated mainly with the precentral gyrus, whereas Str_caudal showed more coactivation with the postcentral gyrus and dorsal cingulate gyrus, which implies that these two subregions’ circuits are responsible for related but distinct functions. More detailed discussions of the functional connectivity pattern for each striatal subregion were presented in the discussion part of **Supplementary Material**.

It should be noticed that insula (BA13) was strongly coactivated with nearly all of the striatum subregions except for Str_occipital and Str_temporal. In Tziortzi and colleagues’ connectivity striatal atlases, probabilistic connections of striatum voxels were related with the frontal, parietal, occipital, and temporal lobes, but not with the insular lobe. However, a meta-analysis of caudate and putamen functional connectivity showed patterns that are consistent with these structural projections, particularly between the putamen and dorsal posterior insula and between the caudate and anterior ventral insula (50). A recent study employing dynamic causal modeling showed that a reward-based attentional effect could be predicted based on changes in spontaneous functional connectivity between the anterior insula and ventral striatum (51), and those findings were confirmed in another functional MRI study involving transcranial magnetic stimulation (52). It is noteworthy that connectivity between insula and striatum had been suspected as the bleeding of the cortical signal into the striatum due to the proximity of the insula to the striatum (21). However, after regressing out the cortical signal within 8 or 9 mm of the striatum, most of the correlations in the posterior putamen and insula were only mitigated but not fully removed. More high-resolution functional imaging work is needed to provide evidence for the relationship between insula and striatum.

Another brain region identified by this MACM study was the thalamus, which was coactivated with almost all striatum subregions. Specifically, the Str_limbic and Str_executive subregions showed functional connectivity with the dorsomedial thalamic nucleus, whereas the Str_rostral-motor, Str_caudal-motor, and Str_parietal subregions showed functional connectivity with the ventral posterior lateral thalamic nuclei. Previous studies demonstrated that the dorsomedial nucleus was highly interconnected with the PFC, which suggests that it may be involved in modulating cognitive functions, emotional reaction, and regulation of alertness, whereas the ventrolateral nuclei have been strongly associated with voluntary movement (53–55). Those findings were also supported by the BD and PC results produced by our MACM. Overall, our results confirm that the thalamus is the common output of the striatum and add new evidence consistent with the corticostriatal-thalamocortical loop theory.

Except for consistent structure connectivity and task-related functional connectivity, results of the task-independent RSFC analysis in healthy subjects also overlapped with those of the task-dependent MACM, providing further evidentiary support for the corticostriatal functional connectivity map. However, the RSFC analysis results were more widely distributed than those of MACM, which may arise from the fundamental differences of the two analytical approaches and the small sample size of healthy controls comparing with bigger Brainmap datasets. In summary, our task-dependent and task-independent functional analysis results using ROIs extracted from corticostriatal structural connectivity atlases showed consistent functional and structural connectivity of corticostriatal circuit, providing a good template that can be extended to the clinical population to examine dysfunctional in corticostriatal circuit.

### Altered Functional Connectivity in FES

Our study identified abnormal functional connectivity in limbic and executive striatum subregions in FES. Specifically, compared with HC, significantly reduced functional connectivity between the limbic subregion and thalamus, mPFC, ACC, IFG, and insula was identified in the FES group. And FES also showed decreased functional connectivity between executive subregions and thalamus, insula, and SMA.

Limbic striatum subregion, on the one hand, presented reduced functional connectivity with some areas in the default mode network (DMN) in schizophrenia, like mPFC and ACC, which are consistent with previous studies. Previous researches suggested DMN is associated with internal cognition and self-related processing and to be deactivated in goal-oriented tasks, and a failure to suppress DMN always leads to impaired performance in tasks (56). A vast of molecular and neuroimaging studies demonstrated the existence of an interaction between striatum and DMN; for example, using PET, Braskie et al. (57) found dopamine synthesis in striatum helped modulate default network activity in working memory task. In recent years, abnormal interaction between striatum and DMN in schizophrenia was identified in other studies. For example, Orliac et al. (58) showed weaker RSFC between DMN and striatal regions in schizophrenia patients; Wang et al. (59) also proved a breakdown of the striatum–DMN loop in schizophrenia using RSFC. Our study further confirms that the dysfunction of striatum–DMN corresponded to limbic subregion. The abnormalities of striatum–DMN not only result in a failure of modulating DMN but also lead to less feedback to the limbic striatum, which further impaired the processing of decision making, working memory, reward, and other cognition that have been observed in schizophrenia.

On the other hand, limbic striatum subregion also showed decreased functional connectivity with the salient network (SN), for example, medial ACC and insula, which are responsible for receiving sensory information from subcortex area and switching from resting state to goal-oriented task state (60). Orliac et al. (58) also reported reduced functional connectivity between striatum and SN in schizophrenia, and the connectivity decrease was correlated with delusion and depression scores. A heuristic framework proposed that altered corticostriatal-thalamic loop resulted from a dysregulated dopamine in striatum, which directly leads to impairments of SN for the attentional allocation to unrelated stimuli. And the schizophrenic individual imposes on these experiences of abnormal salience in an effort to make sense of them and leads to variable phenomenological expression as delusions (61). More evidence is required to directly detect the relationship between limbic striatum subregion and positive symptom in schizophrenia.

Dysfunctional connectivity of executive striatum subregion was mainly focused on thalamus (ventral lateral nucleus) and SMA. Executive striatum subregion constituted of brain areas that have the highest structure connectivity with BA9, BA9/46, and BA10 of the dorsolateral prefrontal cortex related to central-executive network (CEN). Contrary to DMN, CEN was triggered by external goal-oriented task but silence during rest. The identified RSFC between Str_executive and related motor brain areas, such as ventral lateral nucleus in thalamus involving in the motor pathway, as well as SMA associated with the execution of motor (54), suggested that in resting state the brain not only monitors external information but also prepares some motor action. In other words, this functional connectivity is related to flexible and sensitive protection response when sudden stimuli occur. Thus, altered functional connectivity of executive corticostriatal-thalamic in schizophrenia directly leads to reduced inputs from striatum to thalamus, further influences the signal receiving of cortical area like SMA, and eventually results in some symptoms such as slow movement or response in schizophrenia (62). Further investigations are required to check and examine the motor pathway in schizophrenia.

### Limitations

There are several potential methodological limitations in this study. It is important to note that the BOLD imaging method used in all adopted MRI articles was not sensitive enough to detect all the connections between striatum and cortical areas as rigid representations of anatomic connectivity. However, as a meta-analysis, our study did report some steady and reliable connections by combing thousands of articles using functional neuroimaging experiments, which give a supplement for the anatomic connectivity. Another major limitation of this MACM study was the database employed. Even though Brainmap is one of the largest databases of published functional and structural neuroimaging experiments with coordinate-based results, it is not exhaustive. Finally, the relatively small sample size compared with bigger datasets from Brainmap, the statistical method, the analysis threshold, and also the correction method used in present study constituted a limitation of picking up sensitive connections in RSFC analysis.

### Conclusion

Our MACM and RSFC results obtained from task-dependent and task-independent functional connectivity confirm and extend previous findings indicating that the organization of cortical inputs to the striatum is highly ordered into subdivisions of the striatum, especially that of frontal and parietal lobe afferents. More importantly, using this connectivity pattern, we further confirm the reduced functional connectivity between limbic striatum subregions and thalamus and some brain areas in DMN (e.g., mPFC, ACC) and SN (e.g., insula) and also between executive striatum subregions and thalamus, SMA, and insula in FES, which supports the important role of the corticostriatal-thalamic loop in the pathophysiology of schizophrenia.

## Data Availability Statement

The datasets generated for this study are available on request to the corresponding author.

## Ethics Statement

The studies involving human participants were reviewed and approved by the Second Xiangya Hospital of Central South University. The patients/participants provided their written informed consent to participate in this study.

## Author Contributions

XW, BZ, and PL conceived the presented idea and wrote the manuscript, in consultation with DÖ and XSW. BZ analyzed the data with support from PL and XJ, and PL also contributed to the figure presented in the manuscript. WS and SY helped with revision of the article.

## Funding

This work was supported by the National Natural Science Foundation of China (XW, grant 31671144) (PL, grant 61473221), Shanghai Municipal Science and Technology Major Project (grant 2018SHZDZX01), and Hunan Provincial Natural Science Foundation of China (XSW, grant 2019JJ40362).

## Conflict of Interest

The authors declare that the research was conducted in the absence of any commercial or financial relationships that could be construed as a potential conflict of interest.

## Abbreviations

MACM, meta-analytic connectivity modeling; DTI, diffusion tensor imaging; ROIs, regions of interests; RSFC, resting-state functional connectivity; FES, first-episode schizophrenia; DLPFC, dorsolateral prefrontal cortex; PET, positron emission tomography; mPFC, medial prefrontal cortex; BDs, behavior domains; PCs, paradigm classes; ALE, activation likelihood estimation; FDR, false discovery rate; FWE, family-wise error rate; EPI, echo-planar imaging; MNI, Montreal Neurological Institute; BOLD, blood oxygenation level dependent; BA, Brodmann area; MFG, middle frontal gyrus; IFG, inferior frontal gyrus; STG, superior temporal gyrus; SMA, supplementary motor area; ACC, anterior cingulate cortex; Str_limbic, Str_executive, Str_rostral-motor, Str_caudal-motor, Str_parietal, Str_occipital and Str_temporal, striatum subregions of limbic, executive, rostral-motor, caudal-motor, parietal, occipital and temporal; DMN, default mode network; SN, salient network; CEN, central-executive network.
