# Supplementary material for: Altered Functional Connectivity of Striatum Based on the Integrated Connectivity Model in First-Episode Schizophrenia
Source: Front Psychiatry. 2019 Oct 18;10:756. doi: 10.3389/fpsyt.2019.00756 (PMC6813199; doi:10.3389/fpsyt.2019.00756)
Supplement: Supplementary file 1 [file DataSheet_1.docx]

Supplementary Material

**Results**

1. **Task-dependent MACM analysis**

Table S1 MACM results of Str_limbic subregion (p<.01, FDR corrected)

| **Cluster** | **Anatomical Region** | **BA** | **X** | **Y** | **Z** | **Volume mm^3^** | **Maximum ALE Value** |
| --- | --- | --- | --- | --- | --- | --- | --- |
| 1 | L Putamen |  | -14 | 8 | -2 | 64072 | 0.538 |
|  | R Caudate Head |  | 12 | 8 | 0 |  | 0.491 |
|  | R Medial Dorsal Nucleus |  | 6 | -14 | 8 |  | 0.191 |
|  | L Medial Dorsal Nucleus |  | -10 | -18 | 6 |  | 0.177 |
|  | R Insula | 13 | 34 | 18 | 4 |  | 0.170 |
|  | L Insula | 13 | -32 | 18 | 2 |  | 0.151 |
|  | L Precentral Gyrus | 44 | -48 | 4 | 6 |  | 0.089 |
|  | R Hippocampus |  | 28 | -18 | -12 |  | 0.083 |
| 2 | R Cingulate Gyrus | 32 | 2 | 14 | 38 | 7968 | 0.144 |
|  | L Cingulate Gyrus | 24 | 0 | 0 | 46 |  | 0.129 |
|  | L Medial FrontalGyrus | 6 | -2 | -6 | 58 |  | 0.095 |
| 3 | L PostcentralGyrus | 40 | -54 | -22 | 20 | 376 | 0.080 |

Table S2 MACM results of Str_executive subregion (p<.01, FDR corrected)

| **Cluster** | **Anatomical Region** | **BA** | **X** | **Y** | **Z** | **Volume mm^3^** | **Maximum ALE Value** |
| --- | --- | --- | --- | --- | --- | --- | --- |
| 1 | L Putamen |  | -22 | 4 | 6 | 134520 | 0.578 |
|  | L Thalamus |  | -12 | -16 | 8 |  | 0.384 |
|  | R Claustrum |  | 30 | 16 | 6 |  | 0.363 |
|  | L Insula | 13 | -30 | 18 | 6 |  | 0.362 |
|  | R Medial Dorsal Nucleus |  | 8 | -16 | 10 |  | 0.346 |
|  | L Insula | 13 | -48 | 6 | 6 |  | 0.249 |
|  | L Inferior Frontal Gyrus | 9 | -50 | 6 | 24 |  | 0.230 |
|  | L Precentral Gyrus | 6 | -48 | 2 | 32 |  | 0.225 |
|  | L Precentral Gyrus | 4 | -48 | -14 | 38 |  | 0.194 |
|  | R Precentral Gyrus | 6 | 48 | -10 | 38 |  | 0.193 |
|  | L Inferior ParietalLobule | 7 | -30 | -56 | 44 |  | 0.191 |
|  | R Precentral Gyrus | 44 | 46 | 8 | 8 |  | 0.188 |
|  | L Insula | 40 | -48 | -26 | 18 |  | 0.181 |
|  | R Inferior Frontal Gyrus | 9 | 48 | 4 | 30 |  | 0.180 |
|  | L Inferior Parietal Lobule | 40 | -38 | -48 | 42 |  | 0.173 |
|  | L Precentral Gyrus | 4 | -36 | -18 | 54 |  | 0.168 |
|  | L Superior Parietal Lobule | 7 | -24 | -66 | 42 |  | 0.165 |
|  | L Middle Frontal Gyrus | 9 | -44 | 18 | 28 |  | 0.154 |
|  | L Middle Frontal Gyrus | 6 | -30 | -4 | 52 |  | 0.151 |
|  | L Postcentral Gyrus | 40 | -38 | -32 | 50 |  | 0.146 |
|  | L Inferior Parietal Lobule | 40 | -56 | -22 | 28 |  | 0.135 |
|  | L Superior Temporal Gyrus | 41 | -56 | -16 | 8 |  | 0.133 |
|  | L Superior Temporal Gyrus | 42 | -58 | -32 | 8 |  | 0.123 |
| 2 | L Medial FrontalGyrus | 6 | -2 | 2 | 48 | 21808 | 0.394 |
| 3 | R Postcentral Gyrus | 40 | 54 | -22 | 22 | 5160 | 0.151 |
|  | R Inferior Parietal Lobule | 40 | 54 | -38 | 26 |  | 0.140 |
|  | R Superior Temporal Gyrus | 22 | 54 | -28 | 6 |  | 0.139 |
| 4 | L Culmen |  | -26 | -56 | -24 | 3896 | 0.179 |
|  | L Declive |  | -36 | -58 | -16 |  | 0.147 |
| 5 | R Middle Frontal Gyrus | 9 | 32 | 36 | 28 | 3224 | 0.174 |
|  | R Middle Frontal Gyrus | 46 | 42 | 34 | 24 |  | 0.153 |
| 6 | R Culmen |  | 26 | -58 | -24 | 2472 | 0.151 |
|  | R Culmen |  | 24 | -50 | -22 |  | 0.145 |
| 7 | R Inferior Parietal Lobule | 40 | 34 | -48 | 42 | 1920 | 0.162 |

Table S3 MACM results of Str_rostral-motor subregion (p<.01, FDR corrected)

| **Cluster** | **Anatomical Region** | **BA** | **X** | **Y** | **Z** | **Volume mm^3^** | **Maximum ALE Value** |
| --- | --- | --- | --- | --- | --- | --- | --- |
| 1 | L Putamen |  | -24 | -2 | 10 | 14224 | 0.263 |
|  | L VentralPosterior Lateral Nucleus |  | -14 | -16 | 8 |  | 0.104 |
|  | L Insula | 13 | -32 | 16 | 8 |  | 0.080 |
|  | R Precentral Gyrus | 4 | -48 | -12 | 44 |  | 0.070 |
|  | R Superior Temporal Gyrus | 22 | -50 | 4 | 8 |  | 0.069 |
|  | R Inferior Frontal Gyrus | 9 | -54 | 4 | 24 |  | 0.065 |
|  | L Precentral Gyrus | 6 | -52 | 0 | 34 |  | 0.061 |
| 2 | L Putamen |  | 22 | -2 | 10 | 12768 | 0.271 |
|  | L Thalamus |  | 12 | -18 | 8 |  | 0.102 |
|  | L Inferior Frontal Gyrus | 44 | 54 | 6 | 22 |  | 0.094 |
|  | L Claustrum |  | 30 | 16 | 6 |  | 0.077 |
| 3 | R Medial Frontal Gyrus | 6 | -2 | -8 | 58 | 6920 | 0.156 |
|  | L Cingulate Gyrus | 24 | -4 | 4 | 42 |  | 0.071 |
| 4 | L Postcentral Gyrus | 40 | -54 | -24 | 20 | 1408 | 0.078 |
|  | L Inferior Parietal Lobule | 40 | -58 | -22 | 26 |  | 0.068 |
|  | L Postcentral Gyrus | 2 | -52 | -26 | 34 |  | 0.055 |
|  | L Insula | 13 | -48 | -38 | 14 |  | 0.052 |
| 5 | L Precentral Gyrus | 4 | -36 | -16 | 56 | 520 | 0.069 |
| 6 | L Inferior Parietal Lobule | 40 | -36 | -46 | 46 | 432 | 0.057 |

Table S4 MACM results of Str_caudal-motor subregion (p<.01, FDR corrected)

| **Cluster** | **Anatomical Region** | **BA** | **X** | **Y** | **Z** | **Volume mm^3^** | **Maximum ALE Value** |
| --- | --- | --- | --- | --- | --- | --- | --- |
| 1 | L Putamen |  | -26 | -6 | 8 | 16704 | 0.344 |
|  | L Ventral Posterior Lateral Nucleus |  | -14 | -16 | 6 |  | 0.123 |
|  | L Insula | 13 | -46 | 4 | 6 |  | 0.102 |
|  | L Inferior Frontal Gyrus | 9 | -54 | 2 | 22 |  | 0.079 |
|  | L Precentral Gyrus | 6 | -52 | -4 | 38 |  | 0.064 |
| 2 | R Putamen |  | 24 | -8 | 10 | 15376 | 0.200 |
|  | R Thalamus |  | 12 | -16 | 8 |  | 0.114 |
|  | R Claustrum |  | 30 | 14 | 8 |  | 0.077 |
|  | R Inferior Frontal Gyrus | 9 | 54 | 4 | 26 |  | 0.075 |
|  | L Precentral Gyrus | 44 | 46 | 6 | 8 |  | 0.074 |
| 3 | L Medial Frontal Gyrus | 6 | 0 | -8 | 58 | 8952 | 0.168 |
|  | R Cingulate Gyrus | 24 | 2 | 2 | 42 |  | 0.084 |
|  | R Cingulate Gyrus | 24 | -6 | 2 | 42 |  | 0.080 |
| 4 | L Postcentral Gyrus | 3 | -38 | -30 | 50 | 1504 | 0.076 |
|  | L Inferior Parietal Lobule | 40 | -42 | -36 | 40 |  | 0.062 |
| 5 | R Culmen |  | 20 | -50 | -20 | 968 | 0.071 |
|  | L Declive |  | 22 | -60 | -22 |  | 0.066 |
| 6 | R Culmen |  | -26 | -56 | -22 | 816 | 0.075 |
| 7 | L Postcentral Gyrus | 40 | -50 | -26 | 18 | 728 | 0.066 |
|  | L Inferior Parietal Lobule | 40 | -56 | -24 | 24 |  | 0.061 |
| 8 | R Precentral Gyrus | 4 | 34 | -22 | 54 | 416 | 0.064 |

Table S5 MACM results of Str_parietal, Str_occipital and Str_temporal subregions (p<.01, FDR corrected)

| **Cluster** | **Anatomical Region** | **BA** | **X** | **Y** | **Z** | **Volume mm^3^** | **Maximum ALE Value** |
| --- | --- | --- | --- | --- | --- | --- | --- |
| Str_parietal | | | | | | | |
| 1 | L Putamen |  | -28 | -12 | 4 | 11736 | 0.205 |
|  | L Ventral Posterior Medial Nucleus |  | -14 | -18 | 8 |  | 0.071 |
|  | R Caudate Body |  | -14 | -4 | 18 |  | 0.071 |
| 2 | R Putamen |  | 24 | -14 | 8 | 9184 | 0.096 |
|  | R Putamen |  | 20 | 2 | 12 |  | 0.070 |
|  | R Caudate Body |  | 12 | -6 | 20 |  | 0.062 |
|  | R Medial Dorsal Nucleus |  | 10 | -18 | 10 |  | 0.053 |
| 3 | R Cingulate Gyrus | 24 | -2 | 0 | 46 | 5592 | 0.100 |
|  | L Medial Frontal Gyrus | 6 | -4 | -8 | 54 |  | 0.087 |
| 4 | L Dentate |  | 18 | -58 | -20 | 1560 | 0.067 |
| 5 | R Insula | 13 | -44 | -2 | 12 | 696 | 0.060 |
| 6 | L Dentate |  | -18 | -56 | -22 | 344 | 0.049 |
| Str_occipital | | | | | | | |
| 1 | R Lateral Globus Pallidus |  | 26 | -10 | -4 | 1024 | 0.058 |
| 2 | L Putamen |  | -28 | -14 | -4 | 688 | 0.041 |
| Str_temporal | |  |  |  |  |  |  |
| No significant brain areas were activated. | | | | | | | |

1. **Task-independent RSFC analysis**

The RSFC analysis results are summarized in following tables. RSFC analysis indicated that Str_limbic has significant connectivity with a broad frontal areas, including the medial frontal cortex (mPFC), middle frontal gyrus (MFG), right inferior frontal gyrus (IFG), left anterior cingulate cortex (ACC), as well as with the left putamen and right caudate (details in Table S6). Meanwhile RSFC analysis indicated that Str_executive has significant connectivity with the bilateral putamen, left MFG, and left SFG, right mPFC, left supplementary motor area (SMA), right ACC (Table S7).

RSFC analysis showed that Str_rostral-motor related coactivation (Table S8) with brain areas including bilateral SMA, right superior frontal cortex (SFG) (BA6), left superior temporal gyrus as well as the bilateral putamen and right caudate. Meanwhile, Str_caudal-motor (Table S9) was found to associate strongly with activity of the left SMA (BA6), left STG (BA38), and cingulate gyrus (BA24) in the cortex, as well as with the bilateral putamen.

Str_parietal was found to have strong RSFC with the left SMA (BA6), SFG, right mPFC, bilateral precentral gyrus (BA 6/44) and left postcentral gyrus (BA3) as well as with the bilateral putamen (Table S10). Str_occipital was found to have strong RSFC with bilateral IFG (BA44), right SFG (BA6), SMA (BA24), precentral gyrus (BA9), postcentral gyrus, and left iferior parietal lobule as well as with the bilateral putamen (Table S11). Str_temporal was found to have strong RSFC with the bilateral thalamus, right putamen, right caudate, left cingulate gyrus and right amydala (Table S12).

Table S6 RSFC results of Str_limbic in HC (p<.05, FWE corrected)

| cluster | Anatomical Region | BA | X | Y | Z | Volume mm3 | T |
| --- | --- | --- | --- | --- | --- | --- | --- |
| 1 | R Caudate |  | 9 | 9 | -6 | 3141 | 31.48 |
|  | L Putamen |  | -21 | 15 | -6 |  | 29.68 |
|  | L Medial Frontal Gyrus |  | 21 | 12 | -9 |  | 27.20 |
| 2 | R Cingulum_Ant | 32 | 3 | 18 | 21 | 228 | 9.44 |
|  | L Anterior Cingulate | 24 | -6 | 24 | 24 |  | 8.98 |
| 3 | L Middle Frontal Gyrus |  | -27 | 45 | 18 | 32 | 8.00 |
| 4 | R Inferior Frontal Gyrus |  | 33 | 36 | 15 | 10 | 7.89 |
| 5 | L Thalamus |  | -6 | -15 | -3 | 14 | 7.12 |

Table S7 RSFC results of Str_executive in HC (p<.05, FWE corrected)

| cluster | Anatomical Region | BA | X | Y | Z | Volume mm3 | T |
| --- | --- | --- | --- | --- | --- | --- | --- |
| 1 | L Putamen |  | -21 | 6 | 3 | 2841 | 35.80 |
|  | R Caudate |  | 15 | 12 | 9 |  | 22.14 |
|  | R Putamen |  | 27 | 9 | 0 |  | 20.51 |
| 2 | L Frontal_Sup_Medial |  | -9 | 24 | 42 | 208 | 8.58 |
|  | L Cingulum_Ant |  | -6 | 27 | 27 |  | 8.20 |
|  | L Supp_Motor_Area |  | -3 | 9 | 60 |  | 8.05 |
| 3 | R Medial Frontal Gyrus | 10 | 15 | 54 | 0 | 11 | 7.60 |
| 4 | L Superior Frontal Gyrus | 10 | -27 | 48 | 12 | 26 | 6.98 |
|  | L Middle Frontal Gyrus |  | -27 | 36 | 21 |  | 6.96 |
| 5 | L Cingulum_Mid | 24 | -9 | 9 | 33 | 5 | 6.82 |
| 6 | R Anterior Cingulate | 10 | 12 | 33 | 0 | 10 | 6.71 |

Table S8 RSFC results of Str_rostral-motor in HC (p<.05, FWE corrected)

| cluster | Anatomical Region | BA | X | Y | Z | Volume mm3 | T |
| --- | --- | --- | --- | --- | --- | --- | --- |
| 1 | R Putamen |  | 27 | 3 | 6 | 2100 | 31.19 |
|  | L Extra-Nuclear |  | -27 | 0 | -9 |  | 25.18 |
| 2 | R Supp_Motor_Area | 32 | 6 | 6 | 51 | 203 | 8.84 |
|  | L Supp_Motor_Area | 32 | -3 | 6 | 51 |  | 8.07 |
|  | R Superior Frontal Gyrus | 6 | 3 | 3 | 63 |  | 7.61 |
| 3 | R Cingulum_Mid |  | -9 | 9 | 36 | 5 | 6.81 |

Table S9 RSFC results of Str_caudal-motor in HC (p<.05, FWE corrected)

| cluster | Anatomical Region | BA | X | Y | Z | Volume mm3 | T |
| --- | --- | --- | --- | --- | --- | --- | --- |
| 1 | L Putamen |  | -27 | -9 | 9 | 1047 | 26.27 |
| 2 | R Putamen |  | 30 | -3 | -3 | 1052 | 20.44 |
|  | R Caudate |  | 15 | 0 | 18 |  | 14.84 |
|  | R Putamen |  | 30 | -18 | 0 |  | 13.62 |
| 3 | L Supp_Motor_Area |  | -3 | 3 | 51 | 89 | 8.41 |
|  | L Supp_Motor_Area_L |  | 0 | -3 | 63 |  | 8.25 |
|  | L Superior Frontal Gyrus |  | -6 | -18 | 63 |  | 6.52 |
| 4 | L Superior Temporal Gyrus | 22 | -54 | 6 | 0 | 18 | 7.59 |

Table S10 RSFC results of Str_parietal in HC (p<.05, FWE corrected)

| cluster | Anatomical Region | BA | X | Y | Z | Volume mm3 | T |
| --- | --- | --- | --- | --- | --- | --- | --- |
| 1 | L Putamen |  | -30 | -12 | -3 | 2288 | 27.34 |
|  | R Putamen |  | 27 | -15 | 3 |  | 22.63 |
| 2 | L Supp_Motor_Area | 6 | -6 | -9 | 63 | 215 | 8.73 |
|  | R Medial Frontal Gyrus |  | 3 | 0 | 63 |  | 8.70 |
|  | L Superior Frontal Gyrus |  | -3 | 3 | 51 |  | 8.37 |
| 3 | L Postcentral |  | -45 | -9 | 48 | 36 | 8.43 |
|  | L Precentral Gyrus |  | -36 | -9 | 42 |  | 7.75 |
|  | L Precentral | 6 | -51 | -3 | 42 |  | 6.71 |
| 4 | R Precentral | 44 | 57 | 6 | 21 | 53 | 8.01 |

Table S11 RSFC results of Str_occipital in HC (p<.05, FWE corrected)

| cluster | Anatomical Region | BA | X | Y | Z | Volume mm3 | T |
| --- | --- | --- | --- | --- | --- | --- | --- |
| 1 | L Putamen |  | -30 | -12 | -6 | 2006 | 34.34 |
|  | R Putamen |  | 33 | -15 | -6 |  | 20.48 |
| 2 | R Inferior Frontal Gyrus | 44 | 57 | 9 | 18 | 46 | 9.84 |
|  | R Precentral | 9 | 57 | 9 | 33 |  | 7.61 |
| 3 | L Inferior Parietal Lobule |  | -60 | -30 | 24 | 32 | 7.96 |
| 4 | R Supp_Motor_Area |  | 3 | -6 | 45 | 17 | 7.71 |
| 5 | R SupraMarginal |  | 54 | -24 | 30 | 26 | 7.28 |
|  | R Postcentral Gyrus |  | 63 | -21 | 33 |  | 7.10 |
| 6 | R Supp_Motor_Area | 24 | 6 | 6 | 66 | 10 | 7.16 |
|  | R Superior Frontal Gyrus | 6 | 9 | 3 | 57 |  | 6.65 |
| 7 | L Inferior Frontal Gyrus | 44 | -54 | 9 | 15 | 6 | 6.89 |

Table S12 RSFC results of Str_temporal in HC (p<.05, FWE corrected)

| cluster | Anatomical Region | BA | X | Y | Z | Volume mm3 | T |
| --- | --- | --- | --- | --- | --- | --- | --- |
| 1 | R Caudate |  | 9 | 9 | -6 | 1631 | 27.25 |
|  | R Amygdala |  | 24 | 3 | -15 |  | 16.88 |
|  | R Putamen |  | 27 | -12 | -6 |  | 16.28 |
| 2 | L Thalamus |  | -6 | -21 | -3 | 36 | 9.37 |
| 3 | R Thalamus |  | 9 | -21 | -3 | 23 | 8.64 |
| 4 | L Cingulum_Ant |  | -3 | 33 | 0 | 14 | 7.15 |

**Discussion**

1. **Specific task-dependent MACM of striatal subregions**

***Limbic subregion*** Our MACM analysis showed that mPFC/anterior cingulate (BA24/32) and ventrolateral PFC/IFG (BA47) have significant functional connectivity with Str_limbic. The corresponding BD and PC analysis showed those coactivations were related mainly to the emotion and cognition domains, and the performance of reward task paradigms. Previous studies showed that primate (including human) frontal cortex can be divided roughly into three functional areas: the orbital and medial PFC being involved in emotion regulation and motivation, the dorsolateral PFC and ventrolateral PFC being involved in higher cognitive processes or executive functions, and the premotor and motor areas being involved in motor planning and execution (Fuster, 2001; Helmich et al., 2010). The results of this study support this functional division of the frontal cortex. Nevertheless, though the orbitofrontal cortex (OFC) - striatum connection is suggested to be a component of the limbic circuit (Alexander et al., 1986; Alexander et al., 1990), no significant functional connectivity between OFC and Str_limic subregion was detected in our MACM analysis. It could be that the OFC’s proximity to air-filled sinuses makes it particularly susceptible to BOLD sensitivity loss due to susceptibility-induced gradients in the readout direction in typical fMRI EPI sequences (Faro and Mohamed, 2006; Österbauer et al., 2006). Future investigation of this unique structure will benefit from advances in imaging sequences and hardware.

***Executive subregion*** The Str_executive subregion was found to have widespread functional connectivity with the cortex, especially with the fronto-parietal cortex, including the bilateral MFG (BA6/10/46), IFG (BA9), precentral gyrus, SFG (BA4/6), postcentral gyrus, and inferior parietal lobule (BA40). Tziortzi and colleagues found that Str_executive inputs occupied a large portion of the striatum and overlapped with the other subregions connected with the frontal lobe (Str_limbic, Str_rostral-motor, and Str_caudal-motor) (Tziortzi et al., 2014). They therefore suggested that Str_executive and its connectivity with the frontal cortex may be involved in orchestrating the top-down components of decision making, action planning, and selection. In our MACM analysis, the strongest connectivities of the Str_executive subregion were with sites in the dorsolateral PFC (BA9/46), consistent with involvement in higher executive functions and attention allocation. Our BD and PC analysis showed that Str_executive coactivation was related mainly to paradigms that are dependent upon explicit memory and language.

***Rostral-motor and caudal-motor subregions*** Both motor subregions, Str_rostral-motor and Str_caudal-motor, shared similar functional connectivity with the precentral gyrus (BA4/6/44), postcentral gyrus (BA40/2), MFG/dorsal cingulated gyrus (BA24/32), and SMA (BA6). However, Str_rostral-motor coactivated mainly with the precentral gyrus whereas Str_caudal showed more coactivation with the postcentral gyrus and dorsal cingulate gyrus, which implies that these two subregions’ circuits are responsible for related but distinct functions. Previous studies have related the former more closely with motor planning and the latter more closely with motor execution (Braskie et al., 2011; Lim et al., 2011). In our BD analysis, Str_rostral-motor and Str_caudal-motor coactivation were related to the action, cognition, and perception domains. The most strongly associated paradigm for these subregions was finger-tapping, with Str_rostral-motor coactivation also being related to tone monitoring and flexion/extension, whereas Str_caudal-motor coactivation was also related to pain monitoring, repetition, and saccades.

***Parietal subregion*** Unlike the Str_limbic, Str_executive, and Str_motor subregions, the Str_parietal did not show significant functional connectivity with parietal areas or high-level consistency between functional and structural connectivity. However, Str_parietal was found to be functionally associated with the inferior parietal lobule indeed with the threshold at 0.05, FDR corrected (MNI locus 60, -30, 26, BA40). Besides, the MACM analysis results showed that the Str_parietal subregion had significant functional connectivity with the premotor cortex. Although the parietal lobe and premotor cortex are often activated simultaneously during the performance of motor-executive function related tasks, the premotor cortex showed stronger connectivity with Str_parietal than the parietal lobe. MACM showed little connectivity with the Str_occipital and Str_temporal subregions, due at least partly to the dearth of germane studies in the literature (only identified 16 and 4 articles, respectively, in the Brainmap database, as showed in Table 1). It suggested that most previous seed-based studies examining the functional connectivity of the striatum were focused on the frontal or parietal lobe, rather than on the occipital or temporal lobe.

**Reference**

Alexander, G.E., Crutcher, M.D., and DeLong, M.R. (1990). Basal ganglia-thalamocortical circuits: parallel substrates for motor, oculomotor, "prefrontal" and "limbic" functions. *Prog Brain Res* 85**,** 119-146. doi: 0.1016/S0079-6123(08)62678-3.

Alexander, G.E., DeLong, M.R., and Strick, P.L. (1986). Parallel organization of functionally segregated circuits linking basal ganglia and cortex. *Annu Rev Neurosci* 9**,** 357-381. doi: 10.1146/annurev.ne.09.030186.002041.

Braskie, M.N., Landau, S.M., Wilcox, C.E., Taylor, S.D., O'Neil, J.P., Baker, S.L., et al. (2011). Correlations of striatal dopamine synthesis with default network deactivations during working memory in younger adults. *Hum Brain Mapp* 32(6)**,** 947-961. doi: 10.1002/hbm.21081.

Faro, S.H., and Mohamed, F.B. (2006). Functional MRI : basic principles and clinical applications. Springer New York, 261-269.

Fuster, J.M. (2001). The prefrontal cortex--an update: time is of the essence. *Neuron* 30(2)**,** 319-333. doi: 10.1016/S0896-6273(01)00285-9.

Helmich, R.C., Derikx, L.C., Bakker, M., Scheeringa, R., Bloem, B.R., and Toni, I. (2010). Spatial remapping of cortico-striatal connectivity in Parkinson's disease. *Cereb Cortex* 20(5)**,** 1175-1186. doi: 10.1093/cercor/bhp178.

Lim, S.L., O'Doherty, J.P., and Rangel, A. (2011). The decision value computations in the vmPFC and striatum use a relative value code that is guided by visual attention. *Journal of Neuroscience the Official Journal of the Society for Neuroscience* 31(37)**,** 13214-13223. doi: 10.1523/JNEUROSCI.1246-11.2011.

Österbauer, R.A., Wilson, J.L., Calvert, G.A., and Jezzard, P. (2006). Physical and physiological consequences of passive intra-oral shimming. *Neuroimage* 29(1)**,** 245-253. doi: 10.1016/j.neuroimage.2005.07.006.

Tziortzi, A.C., Haber, S.N., Searle, G.E., Tsoumpas, C., Long, C.J., Shotbolt, P., et al. (2014). Connectivity-based functional analysis of dopamine release in the striatum using diffusion-weighted MRI and positron emission tomography. *Cereb Cortex* 24(5)**,** 1165-1177. doi: 10.1093/cercor/bhs397.
